# An Effective Deep Learning Framework for Fall Detection: Model Development and Study Design

**DOI:** 10.2196/56750

**Published:** 2024-08-05

**Authors:** Jinxi Zhang, Zhen Li, Yu Liu, Jian Li, Hualong Qiu, Mohan Li, Guohui Hou, Zhixiong Zhou

**Affiliations:** 1 Beijing Kupei Sports Culture Corporation Limited Beijing China; 2 Institute of Artificial Intelligence in Sports Capital University of Physical Education and Sports Beijing China; 3 School of Physical Education and Sport Science Fujian Normal University Fuzhou China; 4 Institute for Sport Performance and Health Promotion Capital University of Physical Education and Sports Beijing China; 5 Bioelectronics Center of YZW Shanghai China; 6 Walt Technology Group Co, Ltd Jiaxing China

**Keywords:** fall detection, deep learning, self-attention, accelerometer, gyroscope, human health, wearable sensors, Sisfall, MobiFall

## Abstract

**Background:**

Fall detection is of great significance in safeguarding human health. By monitoring the motion data, a fall detection system (FDS) can detect a fall accident. Recently, wearable sensors–based FDSs have become the mainstream of research, which can be categorized into threshold-based FDSs using experience, machine learning–based FDSs using manual feature extraction, and deep learning (DL)–based FDSs using automatic feature extraction. However, most FDSs focus on the global information of sensor data, neglecting the fact that different segments of the data contribute variably to fall detection. This shortcoming makes it challenging for FDSs to accurately distinguish between similar human motion patterns of actual falls and fall-like actions, leading to a decrease in detection accuracy.

**Objective:**

This study aims to develop and validate a DL framework to accurately detect falls using acceleration and gyroscope data from wearable sensors. We aim to explore the essential contributing features extracted from sensor data to distinguish falls from activities of daily life. The significance of this study lies in reforming the FDS by designing a weighted feature representation using DL methods to effectively differentiate between fall events and fall-like activities.

**Methods:**

Based on the 3-axis acceleration and gyroscope data, we proposed a new DL architecture, the dual-stream convolutional neural network self-attention (DSCS) model. Unlike previous studies, the used architecture can extract global feature information from acceleration and gyroscope data. Additionally, we incorporated a self-attention module to assign different weights to the original feature vector, enabling the model to learn the contribution effect of the sensor data and enhance classification accuracy. The proposed model was trained and tested on 2 public data sets: SisFall and MobiFall. In addition, 10 participants were recruited to carry out practical validation of the DSCS model. A total of 1700 trials were performed to test the generalization ability of the model.

**Results:**

The fall detection accuracy of the DSCS model was 99.32% (recall=99.15%; precision=98.58%) and 99.65% (recall=100%; precision=98.39%) on the test sets of SisFall and MobiFall, respectively. In the ablation experiment, we compared the DSCS model with state-of-the-art machine learning and DL models. On the SisFall data set, the DSCS model achieved the second-best accuracy; on the MobiFall data set, the DSCS model achieved the best accuracy, recall, and precision. In practical validation, the accuracy of the DSCS model was 96.41% (recall=95.12%; specificity=97.55%).

**Conclusions:**

This study demonstrates that the DSCS model can significantly improve the accuracy of fall detection on 2 publicly available data sets and performs robustly in practical validation.

## Introduction

According to the World Health Organization, falls rank as the second leading cause of accidental injury-related deaths on a global scale [1]. Statistics from the Centers for Disease Control and Prevention [2] reveal that a significant proportion, at least a quarter, of US residents aged 65 years and older experience a fall annually. Among the older adult population, accidental falls have been the second leading cause of mortality and injury [3]. To prevent falls from causing severe subsequent harm to individuals, it is essential to develop an accurate and efficient fall recognition system, to identify falls and raise the alarm [4]. In the existing research, 2 main categories of systems have emerged: fall prediction systems [5-7] and fall detection systems (FDSs) [8-21].

Although fall prediction systems can assist users in proactively preventing potential falls, the associated installation and maintenance costs can be prohibitively high, thereby limiting its widespread application. In contrast, FDSs can promptly issue alerts upon detecting a fall event, ensuring swift assistance for the individual who has experienced the fall. In recent years, extensive research has been conducted on FDSs and related solutions, categorized as follows: (1) vision-based FDSs [8,9], which monitor and analyze a person’s movements and postures using cameras or imaging devices to detect falls; (2) ambient device–based FDSs [10-12], which use environmental sensors such as Wi-Fi or radar signals to track an individual’s movement data within their living space and detect falls; and (3) wearable sensors–based FDSs [13-21], which use sensors attached to the body to monitor a user’s movements and postures to detect falls. Among these solutions, wearable sensor–based fall detection technology has garnered significant attention because of its affordability and nonintrusive characteristics [21]. Wearable devices, such as inertial measurement units [13,14], smartwatches [15-17], and smartphones [18-20], use high-precision sensors to collect motion data seamlessly. These devices have been widely applied in fall detection and safety monitoring [22].

In wearable sensor–based FDSs, the design of fall detection algorithms is essential. Presently, algorithms used for fall detection can be classified into 3 categories: threshold-based models, machine learning–based models, and deep learning (DL) models. Threshold-based models and machine learning–based models necessitate the extraction of distinctive features from data sets containing fall incidents. In essence, it primarily entails capturing features such as the intensity of activities (eg, magnitude, energy) and variations in the intensity of activities (eg, frequency and SD) from the input data. Threshold-based models discern falls by comparing feature values against predefined thresholds, while machine learning models, such as k-nearest neighbors (KNN) [23], support vector machines (SVM) [24], and decision trees (DT) [25], categorize falls and activities of daily life (ADL) based on the handcrafted features. In contrast, DL models, such as convolutional neural networks (CNN) [26] and long short-term memory (LSTM) [27,28], automatically extract high-level features and scrutinize the temporal characteristics of the data for fall detection.

However, the aforementioned models solely use acceleration data as the input for their algorithms. A previous work [29] has demonstrated that relying exclusively on acceleration data is inadequate for effectively distinguishing falls from ADL. This insufficiency is primarily due to the sensitivity of acceleration data to sensor placement and its inability to capture spatial rotation information. Consequently, researchers advocate for the integration of acceleration data with gyroscope data to provide a more comprehensive understanding of body movements, which can significantly enhance the accuracy of fall detection [30,31].

In this case, Hussain et al [32], Son et al [33], Liu et al [34], and Koo et al [35] use acceleration data with gyroscope data as the input to the model. Hussain et al [32] and Son et al [33] designed fall detection algorithms using KNN and SVM, both of which require manual feature extraction. Additionally, Liu et al [34] developed a CNN-LSTM–based FDS. However, using CNN, LSTM, or their combination fails to evaluate the significance of each component of the feature vector, thereby hindering the differentiation between actual falls and fall-like activities. Moreover, Koo et al [35] proposed a dual-stream–based algorithm for human activity recognition. While this model effectively differentiates between various daily activities, it struggles with the classification of falls and fall-like activities, which exhibit similar data trends.

To address the above challenges, we incorporate the self-attention (SA) mechanism [36] after the CNN module and propose a dual-stream CNN-SA (DSCS) model for fall detection. The SA mechanism has been widely applied in classification tasks such as sleep apnea [37] and skeleton point–based human activity recognition [38]. However, it has not been applied in FDSs. The SA mechanisms empower FDS models to allocate varying weights to different segments of the input data or extracted features.

Compared with existing methods that use manually generated features, the proposed method can automatically extract features using a dual-stream architecture. Specifically, the DSCS model uses acceleration data along with gyroscope data as the input, and then the 2-stream data will pass through a 3-layer CNN to extract discriminable features. Unlike the models using CNN, LSTM, or their combinations, CNN-SA excels at effectively capturing long-term dependencies within input data, enabling the assignment of diverse weights to features from different phases of the fall process. This, in turn, aids the model in achieving an enhanced understanding of the context and pertinent information associated with fall behavior, thereby elevating the model’s capability for contextual modeling.

In this paper, we introduce an accurate and embeddable DSCS model for fall detection. Considering that the handcrafted features rely on expert knowledge and may not yield satisfactory generalization performance, we design a DL framework. In this framework, both accelerometer and gyroscope data are used as input for the model. Then, we introduce a feature-generating method grounded in the CNN-SA architecture. Here, CNN is used to extract features from dual-stream data and capture spatial patterns, to preliminarily identify the pattern differences between fall and ADLs. The feature vector extracted by CNN subsequently passes through an SA layer, which assigns weights to accelerometer and gyroscope features. Finally, the predicted label (fall or ADL) is output by the classification module.

The main contributions of this study are as follows: first, we introduce an accurate and embeddable dual-stream model, DSCS, for fall detection using wearable sensors. The DSCS model comprises a feature extraction module, an SA module, and a classification module. Second, the SA mechanism is applied in the task of fall detection, which assigns different weights to the feature vectors, learning the contribution effect of the sensor data, thereby effectively enhancing classification accuracy. Third, we validate the performance of the DSCS model using publicly available data sets and practical validation. Our model outperforms state-of-the-art machine learning and DL models and demonstrates excellent generalization performance.

The rest of this paper is organized as follows: the *Methods* section illustrates the proposed framework DSCS framework. The *Results* section presents the performance comparison with state-of-the-art algorithms and practical validation. The *Discussion* section covers the results, performance gap, limitations of this study, and conclusions.

## Methods

### Data Sets for Fall Detection

#### Overview

In this paper, we validated the performance of the DSCS model using 2 publicly available data sets: SisFall [39] and MobiFall [40]. The 2 data sets consist of the accelerometer and gyroscope data recorded during fall and ADL trials of multiple participants.

#### SisFall Data Set

The SisFall data set collects accelerometer and gyroscope data during human motion, with a sampling frequency of 200 Hz. This data set comprises data on falls and ADLs collected from 38 volunteers, including 23 young individuals (younger than 30 years) and 15 older individuals (60 years and older). It encompasses 15 falls (such as fall forward, fall backward, and lateral fall) and 19 ADLs (eg, walking, jogging, and jumping).

#### MobiFall Data Set

The MobiFall data set collects accelerometer, gyroscope, and orientation data from smartphones’ built-in inertial measurement units during human motion. The accelerometer has a sampling frequency of 87 Hz, while the gyroscope and orientation sensor have a sampling frequency of 100 Hz. This data set is contributed by 24 volunteers and contains 4 distinct fall activities: fall forward, fall forward with knees on the ground, fall lateral, and fall backward onto a chair. Additionally, it contains 9 various daily activities (eg, standing, walking, and jogging).

#### Data Processing

The processing of accelerometer and gyroscope data consisted of 2 steps: data filtering and data segmentation.

- Data filtering: Considering the susceptibility of sensor data to external environmental noise when worn on the body, we implemented the filtering on the accelerometer and gyroscope data. The filtering process enhances the stability and accuracy of the resulting signals. Since human movements often occur at a frequency of around 20 Hz, a low-pass filter with a cutoff frequency of 20 Hz was used to filter the data [41].
- Data segmentation: A complete fall event typically lasts 8-12 seconds, encompassing 4 phases: prefall, falling, impact, and postfall. To match the sample length in the SisFall and MobiFall data sets, we used the time windows of 12 and 8 seconds for the data segmentation of the SisFall and MobiFall data sets, respectively. Then, we further reduced the sampling rate to 50 Hz to alleviate computational complexity.

### DL Framework

#### Overview

After data processing, we obtained 1798 fall segments and 4117 ADL segments from the SisFall data set and 288 fall segments and 1137 ADL segments from the MobiFall data set. As input to the model, each segment from the SisFall data set has a length of 12 seconds (600 sampling points), while each segment from the MobiFall data set has a length of 8 seconds (400 sampling points). After passing through a DL architecture, each segment produces a predicted label (fall or ADL).

#### Network Architecture

**Figure 1:**
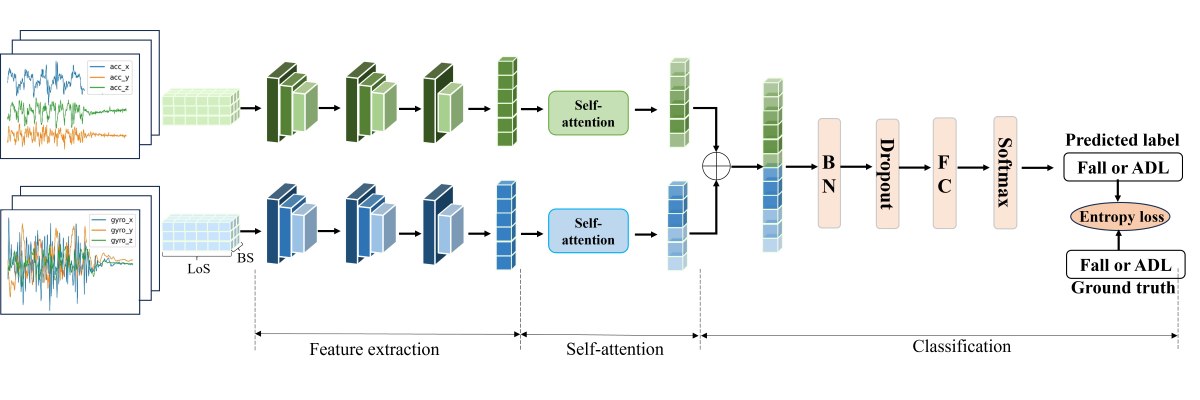
The architecture of the proposed dual-stream convolutional neural network self-attention model. ADL: activities of daily life; BN: batch normalization; BS: batch size; FC: fully connected; LoS: length of segment.

The deep neural network architecture proposed in this paper is illustrated in Figure 1, which mainly consists of 3 modules: feature extraction module, SA module, and classification module.

#### Feature Extraction Module

To extract features, we designed the same architecture for acceleration and gyroscope data stream: a 3-layer CNN to transform the 3-axis data into feature representations. The size of the input data is *N × LoS × 3*, where *N* is the batch size and *LoS* is the length of a segment. The first part of the 3-layer comprises a 1D convolution with 64 filters, a kernel size of 3, and a stride of 1. The outputs of the first 2 layers are activated using a rectified linear unit (ReLU) and followed by a max-pooling layer with a pooling size of 2. The output of the third convolution layer is directly fed into a global pooling layer, which generates a 64D global feature vector for each element in the batch.

#### SA Module

The extracted feature representations from the acceleration and gyroscope data are further refined using the SA module. This module enables the acceleration or gyroscope stream network to efficiently discover the local information highly relevant to discriminating falls and ADL, by capturing the attention weights of local feature information. Specifically, during each training iteration, a total of *N* vectors is fed into the SA module. Within the SA module, these vectors are processed using query, key, and value matrices, each with dimensions of 64×64. Ultimately, a weighted feature vector is generated for each sample in the batch.

#### Classification Module

The weighted acceleration or gyroscope vectors, which have been processed by the SA module, are concatenated to a 128D vector and fed into the classification module. This module is responsible for mapping the concatenated vector to the corresponding prediction label. Specifically, the 128D vector is first processed through a 128D batch normalization (BN) layer, followed by a dropout layer with a dropout probability of 0.5. Finally, the vector is input into a fully connected layer, which comprises a 256D hidden layer and a softmax output layer.

Next, we will provide an in-depth illustration of the architecture and functionality of the 3 modules.

#### Feature Extraction Module

Following Koo et al [35], we design a feature extraction module for accelerometer and gyroscope data streams. This module comprises 3 consecutive encoders, with each encoder combining CNNs and pooling layers.

For acceleration or gyroscope stream, the input data are initially processed by the first encoder, where a 1D CNN extracts shallow-level feature representations from the input data. Subsequently, a max-pooling layer is applied to reduce data dimensions and emphasize key features.

In the second encoder, a CNN further extracts features in a local context, enabling the capture of more intricate patterns and relationships within the time series data. Similarly, a max-pooling layer is used to reduce feature map dimensions and highlight critical features.

The third encoder is dedicated to global feature abstraction, allowing it to capture overarching patterns and essential information across the entire time series, rather than only local features. A global pooling layer enables pooling across the entire feature map, producing a global feature vector that encapsulates summarized information from the entire data sequence.

Through the hierarchical processing by the 3 encoders, the DSCS model can extract both local and global features from the original 3D accelerometer or gyroscope data, thereby enhancing the model’s prediction performance and generalization capabilities.

#### SA Module

The feature vectors generated by the feature extraction module pass through a SA module. This module uses an attention mechanism to dynamically adjust the weights across different positions of the 1D feature vectors, capturing the interdependencies and correlations between different positions.

**Figure 2:**
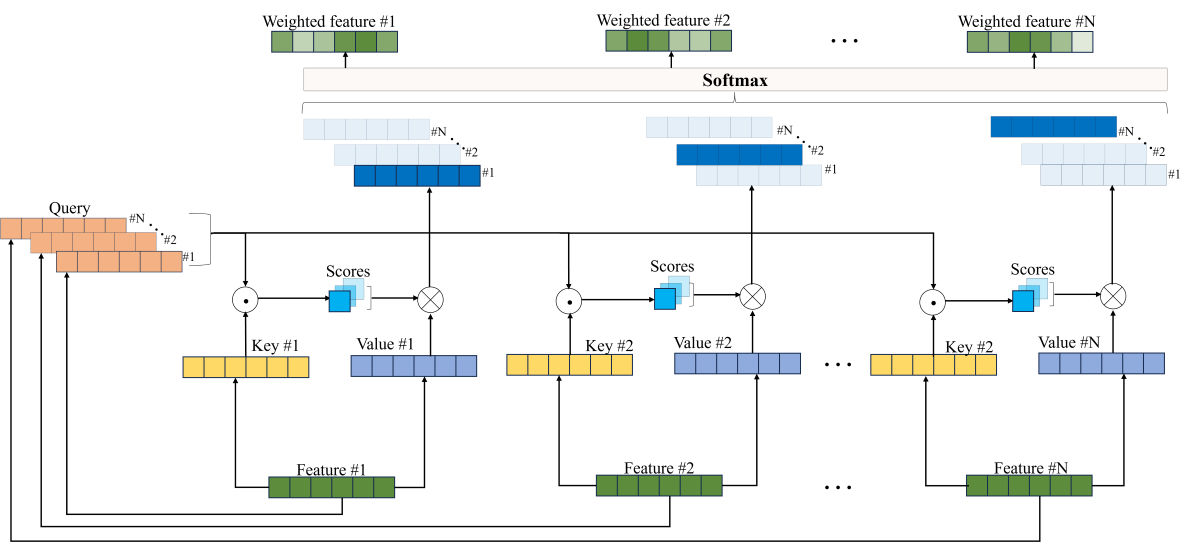
The process of generating weighted feature vectors from original feature vectors.

The detailed process of generating a weighted feature vector is illustrated in Figure 2. Specifically, the SA module constructs query, key, and value vectors by multiplying the input feature vectors with respective weight matrices. The module calculates the dot product between the query and key vectors and then applies the softmax function to determine the relationships between the feature values at each position and those at other positions. The result of the dot product represents the strength of their relationships, commonly referred to as “attention”. Finally, the weighted feature representations for each position are obtained by applying the corresponding attention weights to the value vectors. In the SA mechanism, this weighting operation helps describe the correlations and associations between each position and the others, enabling the model to focus on different positions within the information.

#### Classification Module

The classification module is used to transform the input-weighted feature vectors into the final prediction labels. Specifically, each vector first passes through the BN module. The main function of the BN module is to normalize input data, accelerate model training, and improve the model’s adaptability to different features. Next, the dropout layer is deployed, which randomly sets the output of a portion of neurons to 0, to reduce the risk of overfitting and improve the model’s generalization ability. After BN and dropout, the feature vectors pass through a fully connected layer. Finally, the output of the fully connected layer passes through the softmax layer and generates the probability distribution of fall or ADL labels. The final predicted label generated by the model corresponds to the category with the highest probability.

#### Training Parameters

For each data set, we divided the participants in the data set into training and testing groups with a ratio of 0.8 and 0.2, respectively. The proposed model was deployed in Python (version 3.8.10; Python Software Foundation) using the DL library of *PyTorch* (version 1.7.0). The training process was carried out on a computer server equipped with an Intel Xeon Gold 6330 CPU at 2.00 GHz and an NVIDIA GeForce RTX 3090. We used the cross-entropy loss function and used the Adam optimizer for optimizing the network parameters. In the training phase, we used a maximum of 300 iterations with an initial learning rate set at 0.001, gradually decreasing as the training progressed. The batch size was configured at 128 for optimal model refinement. The hyperparameter selection method for training the model is a random search. Parameters are randomly selected from the hyperparameter space, such as batch size values of 32, 64, and 128, and learning rate values of 0.001, 0.005, and 0.01. By exploring various combinations of batch sizes and learning rates, the optimal hyperparameters are identified based on the performance metrics. This approach ensures that the most effective combination is chosen to achieve the best model performance.

### Performance Evaluation Metrics

To evaluate the fall detection performance of the proposed DSCS model, we used accuracy, recall, precision, and *F*_1_-score as the evaluation metrics. Accuracy is the ratio of the number of segments that are correctly predicted (including falls and ADL) to the total number of segments, which is used to measure the overall performance of the model. Recall is the ratio of the number of correctly predicted fall segments to the total number of fall segments, and high recall means that the model has a strong ability to detect falls. Precision is the ratio of the number of correctly predicted fall segments to the total number of predicted fall segments. High precision means that the model can reduce the number of false alarms and accurately distinguish falls from fall-like activities. *F*_1_-score is calculated based on recall and precision: *F_1_-score = 2 × precision × recall / (precision + recall)*. In practical validation, we also used specificity to evaluate the model’s performance in accurately distinguishing falls from ADLs, including fall-like activities. Specificity is defined as the ratio of correctly predicted ADL segments to the total number of ADL segments.

### Ethical Considerations

The studies involving human participants were reviewed and approved by the Ethics Committee of the Capital University of Physical Education and Sports, Beijing, China (approval number 2023A036). The participants provided their written informed consent to participate in this study.

## Results

### Detection Performance

The performance of the proposed DSCS model was evaluated using the publicly available SisFall and MobiFall data sets. The model demonstrated robust performance on both data sets, achieving an accuracy of 99.32% (recall=99.15%; precision=98.58%; *F*_1_-score=98.86%) on the testing sets of SisFall and an accuracy of 99.65% (recall=100%; precision=98.39%; *F*_1_-score=99.19%) on the testing sets of MobiFall.

**Figure 3:**
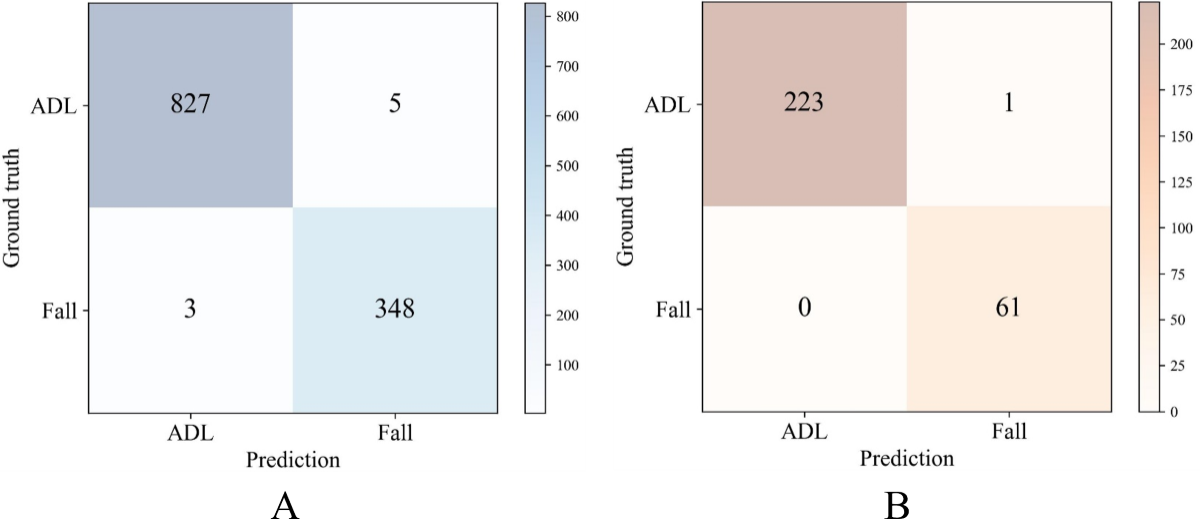
Detection performance of the proposed DSCS model: (A) confusion matrix of SisFall test set and (B) confusion matrix of MobiFall test set. ADL: activities of daily life; DSCS: dual-stream convolutional neural network self-attention.

Figure 3 illustrates the confusion matrices for the DSCS model across 2 test sets: SisFall and MobiFall. Notably, out of 1183 test samples from the SisFall data set, only 8 samples were misclassified (5 false positives and 3 false negatives). Similarly, among the 285 test samples from the MobiFall data set, only 1 sample was misclassified (1 false positive). The result shows that the model can achieve high classification accuracy on both data sets.

### Cross-Validation

To further validate the generalization ability and reliability of the proposed model, we conducted cross-validation to thoroughly assess its performance. The results in Tables 1 and 2 indicate that the proposed model maintains stable performance on both the SisFall and MobiFall data sets. Specifically, the average *F*_1_-scores for 5-fold cross-validation were 99.09% and 98.69%, respectively, while for 10-fold cross-validation, the average *F*_1_-scores were 99.03% and 98.67%, respectively.

**Table 1:** F1-score of the proposed dual-stream convolutional neural network self-attention model under 5-fold cross-validation.

Data set	Fold 1 (%)	Fold 2 (%)	Fold 3 (%)	Fold 4 (%)	Fold 5 (%)	Average (%)
SisFall	99.29	99.01	99.57	98.86	98.72	99.09
MobiFall	99.19	98.36	98.33	98.39	99.17	98.69

**Table 2:** F1-score of the proposed dual-stream convolutional neural network self-attention model under 10-fold cross-validation.

Data set	Fold 1 (%)	Fold 2 (%)	Fold 3 (%)	Fold 4 (%)	Fold5 (%)	Fold 6 (%)	Fold 7 (%)	Fold 8 (%)	Fold9 (%)	Fold 10 (%)	Average (%)
SisFall	99.15	99.43	99.43	99.44	99.72	99.15	99.43	98.86	98.58	99.15	99.03
MobiFall	98.36	100	98.31	98.31	100	98.36	96.67	100	98.36	98.31	98.67

### Comparison With State-of-the-Art Algorithms

We compared our proposed DSCS model with state-of-the-art fall detection models. Specifically, machine learning models—including KNN [32], SVM [33], DT [25]—and DL models—such as CNN [26], LSTM [27], CNN-LSTM [34], contrastive accelerometer-gyroscope embedding [35], few-shot transfer learning [42], and deep convolutional LSTM (DeepConvLSTM) [43]—were used as benchmark models. The results in Table 3 demonstrate the superior performance of the DSCS model. On the SisFall data set, the DSCS model achieved the second-best performance, only slightly behind KNN, which, however, required manual feature extraction. On the MobiFall data set, the DSCS model outperformed all state-of-the-art machine learning and DL models in terms of accuracy, recall, precision, and *F*_1_-score.

**Table 3:** Performance comparison of the proposed DSCS^a^ model with state-of-the-art fall detection algorithms.

Data set, algorithms, and corresponding models				Accuracy (%)		Recall (%)		Precision (%)		*F*_1_-score (%)
**SisFall**										
	**ML^b^**									
		DT^c^ [25]	97.97		96.30		96.85		96.57	
		KNN^d^ [32]	99.41		99.43		98.59		99.01	
		SVM^e^ [33]	99.24		98.86		98.58		98.72	
	**DL^f^**									
		CNN^g^ [26]	97.97		97.44		95.80		96.61	
		LSTM^h^ [27]	97.80		96.01		96.56		96.29	
		CNN-LSTM [34]	98.73		97.15		98.55		97.85	
		CAGE^i^ [35]	98.90		98.29		98.01		98.15	
		FSTL^j^ [42]	98.65		97.44		97.99		97.71	
		DeepConvLSTM^k^ [43]	98.99		98.58		98.02		98.30	
		DSCS	99.32		99.15		98.58		98.86	
**MobiFall**										
	**ML**									
		DT [25]	94.74		95.08		82.86		88.55	
		KNN [32]	98.60		96.72		96.72		96.72	
		SVM [33]	96.14		90.16		91.67		90.91	
	**DL**									
		CNN [26]	95.79		88.52		91.53		90.00	
		LSTM [27]	94.74		85.25		89.66		87.39	
		CNN-LSTM [34]	98.60		96.72		96.72		96.72	
		CAGE [35]	98.95		98.36		96.77		97.56	
		FSTL [42]	97.19		95.08		92.06		93.55	
		DeepConvLSTM [43]	98.25		95.08		96.67		95.87	
		DSCS	99.65		100		98.39		99.19	

^a^DSCS: dual-stream convolutional neural network self-attention.

^b^ML: machine learning.

^c^DT: decision tree.

^d^KNN: k-nearest neighbors.

^e^SVM: support vector machine.

^f^DL: deep learning.

^g^CNN: convolutional neural network.

^h^LSTM: long short-term memory.

^i^CAGE: contrastive accelerometer-gyroscope embedding.

^j^FSTL: few-shot transfer learning.

^k^DeepConvLSTM: deep convolutional LSTM.

### Effect of Low Sampling Rate

To validate the performance of the DSCS model under low sampling rates, we reduced the sampling rate of acceleration and gyroscope data to 10 Hz and 5 Hz and tested the performance of the DSCS model. Table 4 presents the overall performance comparison of the DSCS model under the original sampling rate of 50 Hz and the reduced sampling rates of 10 Hz and 5 Hz. The results show that the accuracy decreased under a sampling rate of 10 Hz and further declined under a sampling rate of 5 Hz. These findings indicate that the variation in sampling rates significantly impacts the accuracy of fall detection.

**Table 4:** Performance comparison of the proposed DSCS^a^ model under different sampling rates.

Data set and model		Accuracy (%)	Recall (%)	Precision (%)	*F*_1_-score (%)
**SisFall**					
	DSCS	99.32	99.15	98.58	98.86
	DSCS (10 Hz)	97.63	96.58	95.49	96.03
	DSCS (5 Hz)	95.60	92.88	92.35	92.61
**MobiFall**					
	DSCS	99.65	100	98.39	99.19
	DSCS (10 Hz)	96.49	95.08	89.23	92.06
	DSCS (5 Hz)	94.04	86.89	85.48	86.18

^a^DSCS: dual-stream convolutional neural network self-attention.

### Effect of SA Mechanism

**Figure 4:**
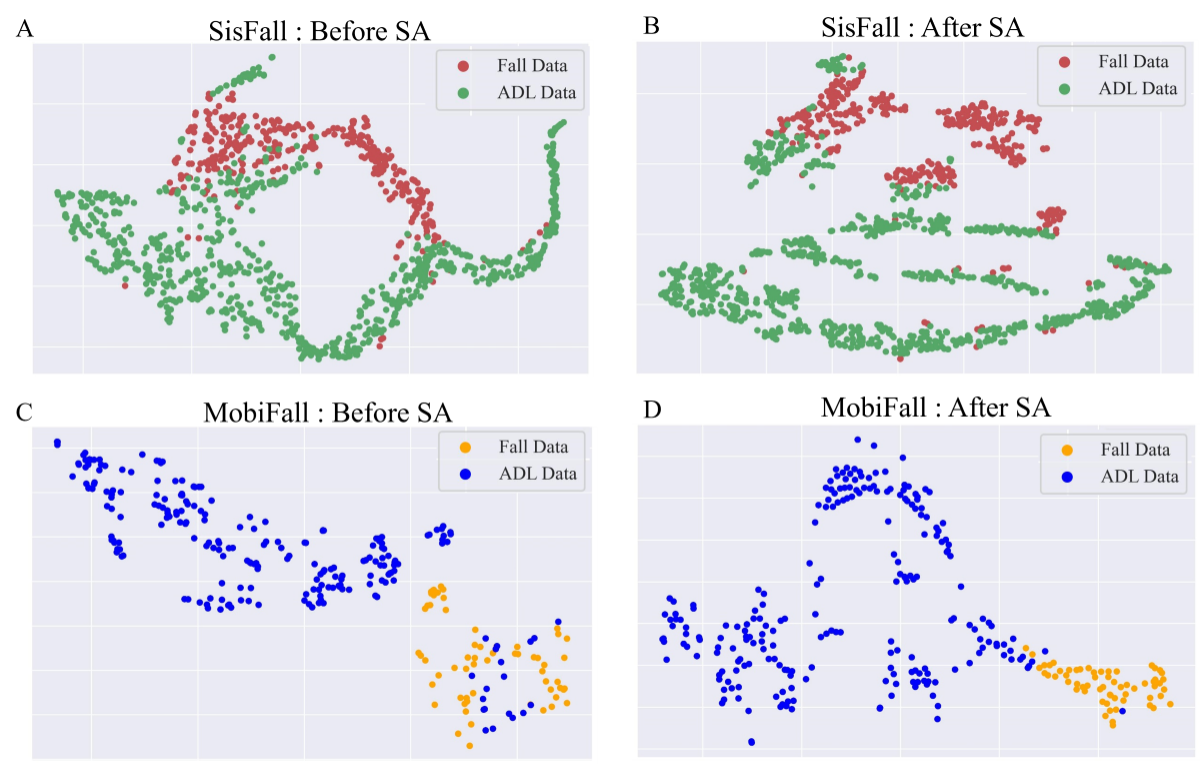
t-SNE plot of (A) SisFall: before SA, (B) SisFall: after SA, (C) MobiFall: before SA, and (D) MobiFall: after SA. ADL: activities of daily life; SA: self-attention; t-SNE: t-distributed stochastic neighbor embedding.

To further explore the data distribution of feature vectors before and after passing through the SA module, we used the t-distributed stochastic neighbor embedding (t-SNE) algorithm for visualizing the test sets from SisFall and MobiFall. The t-SNE algorithm uses nonlinear dimensionality reduction to map the original 64D feature vectors to a more intuitive 2D space while preserving the similarity relationships among data points. The resulting visualization of data distribution through t-SNE allows us to assess the clustering and dispersion of different data categories (fall or ADL) in the 2D space, providing a better understanding and comparison of the separation between fall and ADL. As shown in Figure 4, after passing through the SA module, the feature vectors of different categories became more dispersed. This illustrates why the proposed DSCS algorithm performs better in fall detection and further confirms the effectiveness of the introduced SA mechanism.

### Practical Validation of DSCS

**Figure 5:**
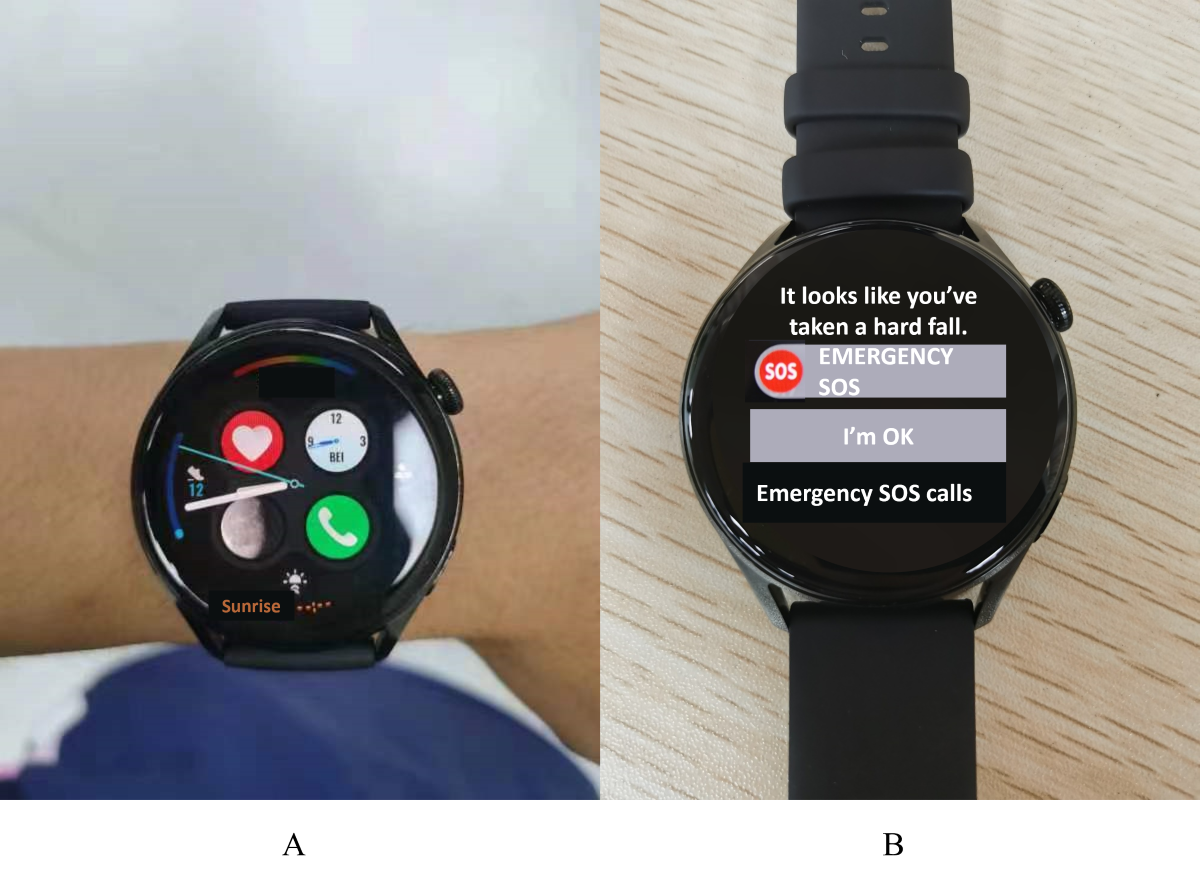
Illustration of practical validation details. (A) Each participant wore a watch. (B) Watch alarm page: prompting detection of fall behavior.

Beyond publicly available data sets, we conducted practical validation on the DSCS model, to assess its generalizability to new data and new users. Specifically, we embedded the DSCS model onto a smartwatch (Huawei Watch 3, Huawei Tech Co, Ltd) equipped with accelerometers and gyroscopes and developed a fall detection alert application. The sensors in the smartwatch operated at a sampling rate of 50 Hz. As depicted in Figure 5A, participants wore smartwatches during experiments. Every 0.5 seconds, the data from the past 8 seconds (400 sampling points) was fed into the fall detection algorithm. When the algorithm detected a fall, a fall alert page, as shown in Figure 5B, was triggered.

**Figure 6:**
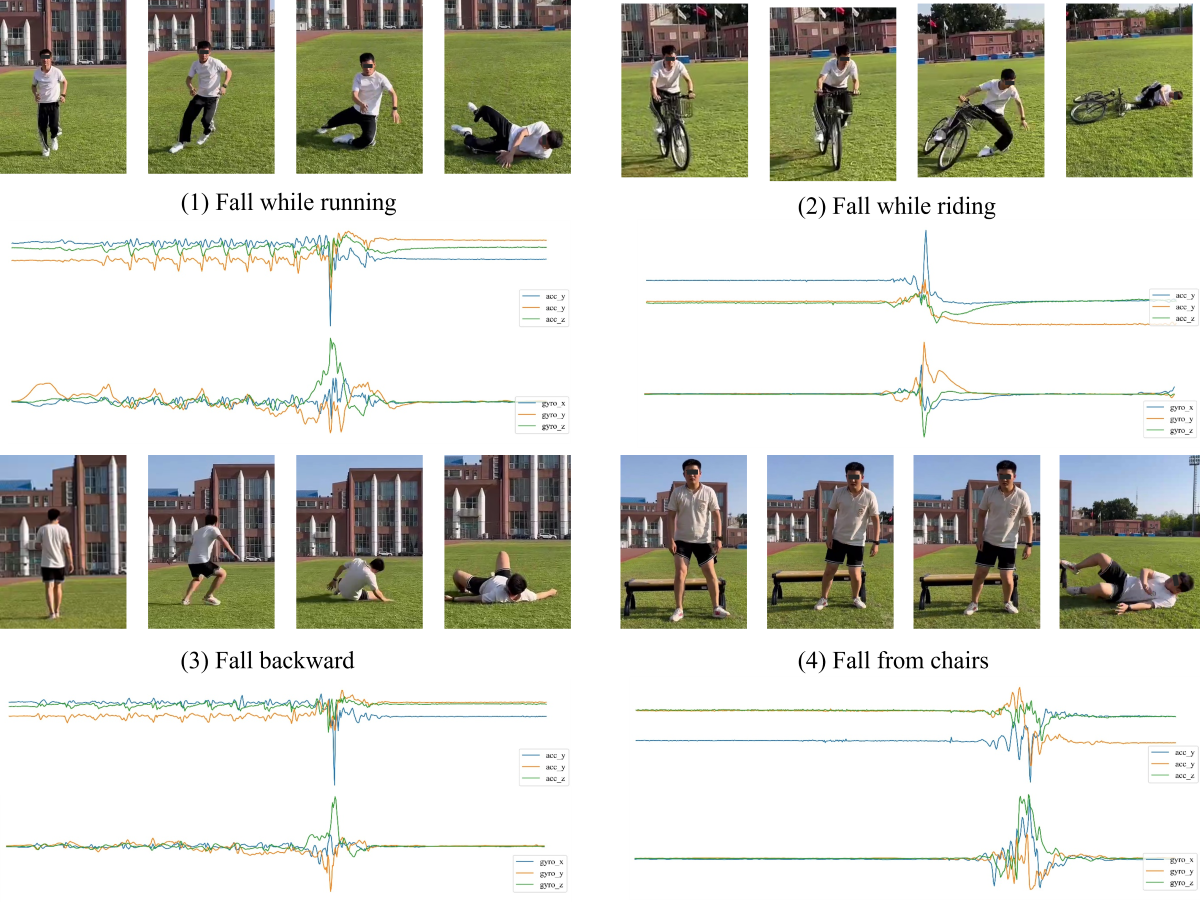
The 4 typical fall activities performed by the participants.

We recruited 10 healthy students from the Capital University of Physical Education and Sports, Beijing, China as participants. The demographics of the participants are detailed in Table 5. Each participant wore a smartwatch and performed 8 fall activities and 9 fall-like activities. The eight fall activities were as follows: (1) fall forward, (2) fall backward, (3) fall to the left, (4) fall to the right, (5) fall from chairs, (6) fall while walking, (7) fall while running, and (8) fall while riding. The nine fall-like activities were as follows: (1) walking, (2) jogging, (3) sprinting, (4) standing and then sitting heavily in a chair, (5) bending down to tie shoelaces, (6) stretching and dropping hands, (7) long jump, (8) descending stairs, and (9) free fall on a trampoline. Figure 6 depicts participants wearing a smartwatch while performing fall activities during the validation process.

**Table 5:** Basic information of the 10 participants.

Information	Participant									
	1	2	3	4	5	6	7	8	9	10
Age (years)	24	25	24	25	35	26	25	26	27	35
Sex	Female	Male	Male	Female	Male	Male	Male	Female	Male	Male
Height (cm)	170	175	183	168	177	180	179	168	178	182
Weight (kg)	53	65	92	70	75	76	75	55	70	80

Each activity was repeated in 100 trials, resulting in a total of 1700 trials (800 fall trials and 900 fall-like trials). The DSCS model achieved satisfactory performance in practical validation, with accuracy, recall, and specificity of 96.41%, 95.12%, and 97.55%, respectively. Table 6 illustrates the recall performance for fall activities and the specificity performance for fall-like activities. The experimental results indicate that the DSCS model maintains a robust fall detection performance in practical applications and possesses satisfactory generalization ability.

**Table 6:** Performance validation of different falls and fall-like activities of daily life.

Performance validation and activity		Recall (%)	Specificity (%)
**Recall performance**			
	Fall forward	97	—^a^
	Fall to the left	96	—
	Fall to the right	96	—
	Fall from chairs	96	—
	Fall while running	96	—
	Fall while walking	94	—
	Fall while riding	93	—
	Fall backward	93	—
**Specificity performance**			
	Descending stairs	—	100
	Stand and then sit heavily in a chair	—	100
	Jogging	—	100
	Walking	—	100
	Bend down to tie shoelaces	—	99
	Stretch and drop hands	—	97
	Long jump	—	95
	Sprinting	—	95
	Free fall on a trampoline	—	92

^a^Not applicable.

## Discussion

### Principal Findings

In this paper, we proposed a DSCS model for fall detection. The proposed model not only performs well on public data sets: SisFall and MobiFall, but also excels in practical validation. By comparing the performance of fall recognition results, we found that the dual-stream mechanism can effectively improve the accuracy of classification. At the same time, from the visualization results of data distribution, it can be observed that after passing through the SA module, the distance between fall data and ADL data becomes larger, which is beneficial for accurately distinguishing falls and ADLs.

### Comparison With Prior Work

Unlike previous studies, this paper integrates the dual-stream data processing architecture and SA module for the first time. Compared with other DL schemes, such as CNN, LSTM, CNN-LSTM, contrastive accelerometer-gyroscope embedding, few-shot transfer learning, and DeepConvLSTM, the proposed scheme in this paper can automatically focus on the features closely related to fall behavior, to achieve better classification performance. In Wang et al [44], the attention mechanism is introduced into the fall detection algorithm. However, in the feature extraction stage, it still uses a machine learning algorithm for manual extraction. Comparative research shows that on the SisFall data set, the proposed model achieved the second-best accuracy (only second to KNN); on the MobiFall data set, the proposed model achieved the best accuracy, recall, and precision.

### Performance Gap Between Public Data Sets and Practical Validation

Beyond public data sets, we embedded the proposed model into smartwatches and developed fall detection and alarm software to verify the fall detection algorithm proposed in this paper in practical applications. According to experimental results, we found that the performance of the model in practical validation dropped, but it was still at a good level. Specifically, accuracy degraded to 96.41%, with recall and specificity being 95.12% and 97.55%, respectively. The main reasons for the performance degradation are differences in sensor models and sampling frequencies, differences in participant conditions, and variations in participant movement norms.

### Limitations

Our research has a few limitations. First, due to practical constraints in data collection, the training and test data sets used in this study may not encompass all possible fall poses, introducing uncertainty regarding the model’s performance in the presence of unique or novel fall scenarios. Second, for safety considerations, the exclusion of older adult data from both public data sets and actual validations during the training and testing of fall detection may compromise the model’s accuracy in handling falls among older adults, who constitute the most vulnerable demographic.

### Conclusions

In this paper, we present the DSCS model, a DL framework designed for fall detection. The DSCS uses a dual-stream architecture incorporating both acceleration and gyroscope data, followed by a 3-layer CNN, SA mechanism, and classification modules. The DSCS model outperforms state-of-the-art algorithms, achieving detection accuracies of 99.32% and 99.65% on the SisFall and MobiFall data sets, respectively. Furthermore, the model maintains a high practical validation accuracy of 96.41%. These results demonstrate the effectiveness of the CNN-SA architecture for classification tasks. This study also highlights how incorporating SA into FDSs can improve classification accuracy by focusing on critical segments of data that significantly contribute to distinguishing between falls and fall-like activities.

## Abbreviations

ADL: activities of daily life
BN: batch normalization
CNN: convolutional neural network
DeepConvLSTM: deep convolutional LSTM
DL: deep learning
DSCS: dual-stream convolutional neural network self-attention
DT: decision tree
FDS: fall detection system
KNN: k-nearest neighbor
LSTM: long short-term memory
ReLU: rectified linear unit
SA: self-attention
SVM: support vector machine
t-SNE: t-distributed stochastic neighbor embedding
